# Myopia control efficacy of second-generation defocus incorporated multiple segments spectacle lenses on fast progressing myopes: Study protocol of a randomised controlled trial

**DOI:** 10.1371/journal.pone.0335061

**Published:** 2025-10-31

**Authors:** Ying Hon, Rachel Ka Man Chun, Tsz Wing Leung, Hua Qi, Keigo Hasegawa, Ka Yan Leung, Chi Ho To, Carly Siu Yin Lam, Dennis Yan-Yin Tse

**Affiliations:** 1 Centre for Myopia Research, School of Optometry, The Hong Kong Polytechnic University, Kowloon, Hong Kong, China; 2 Research Centre for SHARP Vision, The Hong Kong Polytechnic University, Kowloon, Hong Kong, China; 3 InnoHK, Centre for Eye and Vision Research, 17W Hong Kong Science Park, Shatin, Hong Kong, China; 4 Technical Research and Development Department, Vision Care Section, Hoya Corporation, Tokyo, Japan; National Yang Ming Chiao Tung University Hospital, TAIWAN

## Abstract

**Background:**

Spectacle-based interventions for myopia control are appealing to parents and children due to their non-invasive nature. However, long-term efficacy results remain modest and do not account for high-risk children with early-onset myopia and fast progression. This paper presents the protocol of a trial designed to evaluate the efficacy of the new-generation Defocus Incorporated Multiple Segments (DIMS) spectacle lenses in slowing the progression of myopia in children with early-onset and fast myopia progression.

**Methods and design:**

This is a prospective, double-masked, active-controlled, randomised trial (ClinicalTrials.gov identifiers: NCT05888792 and NCT05888805). Participants are Chinese schoolchildren aged 4–12 years with myopia of at least −0.75 diopter (D) in both eyes and with fast progression (≥ 0.50 D per year) or fast axial growth (≥ 0.27 mm per year) in either or both eyes. They are age-stratified and randomly assigned to an experimental arm, a control arm or an auxiliary arm in a 1:1:1 ratio. The experimental arm receives new-generation DIMS spectacle lenses, while the control arm receives single-vision spectacle lenses. The control subjects will crossover to experimental lenses at the end of the first year, and all subjects will continue wearing experimental lenses in the second year. The auxiliary arm receives marketed DIMS spectacle lenses for two years. The primary and secondary outcome measures are the changes in cycloplegic objective refraction and axial length at 12 months from baseline. Peripheral refraction and choroidal thickness will also be monitored, and their relationships with myopia control efficacy will be explored.

**Discussion:**

This study will provide insights into the efficacy of a new generation of DIMS technology for controlling myopia in children with early-onset and fast myopia progression, offering evidence-based practice for myopia management.

**Trial registration:**

ClinicalTrials.gov identifiers: NCT05888792 and NCT05888805

## Introduction

Myopia not only affects school performance, productivity, and quality of life, but also increases the risk of developing blinding ocular pathologies later in life [1]. This was noted in Singapore, in which myopic macular degeneration was found in 65% of elderly participants with high myopia, but was also observed in 31% of those with low myopia [2]. This indicated that there is no safe threshold level of myopia [3]. With the growing prevalence of myopia worldwide, the public health burden due to myopia-related visual impairment is enormous. If nothing is done to combat this trend, it is projected that nearly half of the world’s population (i.e., 4.8 billion) will be myopic by 2050 [4].

Epidemiological evidence indicates an urgent need to prevent and control myopia at the lowest level. Optical interventions to slow myopia progression in children have achieved some success. They include multifocal soft contact lenses, orthokeratology, multifocal, and peripheral defocus spectacle lenses [5]. Of these approaches, spectacles are a favored choice for parents and children due to their minimal side effects and non-invasive nature. The Hong Kong Polytechnic University (PolyU) and Hoya Vision Care jointly developed Defocus Incorporated Multiple Segments (DIMS) technology, which is a pioneer lens design that applies simultaneous central vision correction and peripheral myopic defocus for inhibiting myopia progression. Hundreds of honeycomb-shaped lens segments are uniformly applied onto the spectacle lens, except in the central zone. A two-year randomised clinical trial (RCT) demonstrated DIMS spectacle lenses reduced myopia progression by 52% and axial elongation by 62% in children aged 8–13 years [6]. However, there is currently no treatment that can fully arrest myopia progression in children. Inspired by previous studies that suggested a dose-dependent effect of myopic defocus on myopia control efficacy using multifocal contact lenses [7], progressive [8,9], and peripheral defocus spectacle lenses [10], PolyU and Hoya have developed a new generation of DIMS (D2) technology. The aim of this study is to evaluate the myopia control efficacy of D2 spectacle lenses.

One of the limitations of the previous myopia control trial is the narrow age range of participants, which was restricted to those aged 8–13 years [6]. It has been reported that children with early-onset myopia are prone to faster progression [11,12] and a higher degree of myopia [13,14] in adulthood, making them a high-risk group that requires closer attention. Currently, it remains unclear how children with early-onset myopia and fast progressing myopia benefit from myopia control [15]. Thus, these high-risk cohorts will be recruited in the current study.

This paper describes the protocol of a trial evaluating the efficacy of the D2 spectacle lenses in early-onset and fast-progressing myopes.

## Materials and methods

### Study design and setting

This is a single-site, double-masked, active-controlled, randomised trial with a 1-year duration and a follow-up in the second year. The study comprises a 1-year RCT with an experimental arm, a control arm, and an auxiliary arm. Participants will be randomly allocated in a 1:1:1 fashion to one of the three arms at baseline. Subjects in the experimental and control arms will be assigned to wear D2 and single-vision spectacle lenses, respectively. Subjects in the control arm will crossover to the experimental arm at the end of the first year and all subjects will continue to be followed during the second year. Subjects in the auxiliary arm will be assigned to wear marketed DIMS spectacle lenses for two years, with their myopia control efficacy in early-onset and fast progressing myopes also being investigated. The study is conducted at the Centre for Myopia Research, School of Optometry, PolyU. The SPIRIT guidelines for reporting were followed for this trial protocol. Participant recruitment commenced on 01/09/2023 and is expected to be completed by 01/06/2025.

### Ethics

The study will be conducted in accordance with Good Clinical Practice principles and practices with reference to the requirements for clinical trials with medical devices of the U.S. Food and Drug Administration. The protocol was reviewed and approved by the Institutional Review Board (IRB) of PolyU (HSEARS20221223001–09). The current protocol paper is written based on protocol version 6 dated 10 July 2025.

### Inclusion criteria

The study is enrolling Chinese children aged between 4 and 12 years, with myopia in spherical equivalent refractive error (SER) of at least −0.75D in both eyes and a history of fast progression over the past 2 years. It is recognized that the rate of myopia progression varies with age and gender in children; however, for clinical trial recruitment, a practical approach is adopted by applying a single threshold for fast progression. Fast progression is defined as myopia progression (in SER) at a rate of 0.50D per year or more, or an axial growth rate of 0.27 mm per year or more in either or both eyes. Past prescription forms of the participants issued by registered optometrists in the two years prior to baseline will be considered valid for the calculation of the progression rate.

Children aged 4–6 years old with early onset myopia are at risk of fast myopia progression and high myopia in later adulthood. However, this age group is often identified by optometrists during their very first eye examination. Taking this into account, 4- to 5-year-olds with myopia of at least −0.75D and 6-year-olds with myopia of at least −1.25D in one or both eyes at baseline are also included in the study. The cutoffs are selected based on the age-matched refractive status below the 3^rd^ percentile of refraction in the Chinese population [13].

The distance best-corrected visual acuity of participants should achieve 0.20 logarithm of the minimum angle of resolution (logMAR) or better for those aged 4–6 years, and 0.00 logMAR or better for those aged 7–12 years. Anisometropia should not exceed 1.50D, and astigmatism should be less than or equal to 2.00D.

### Exclusion criteria

- Existing or past eye diseases or surgeries that may have an impact on vision or visual development
- Binocular vision problems
- Long-term medication (intake at least 3 days/week)
- Medication or supplements that affect eye growth
- Systemic diseases that may have an impact on vision or visual development
- Previous or current treatment for myopia control
- Allergies to topical anaesthetic or cyclopentolate eye drops
- Individuals who, in the judgment of the Investigator, are unable to cooperate and follow instructions during eye examination.

### Sample size

Based on a similar RCT conducted previously [6], it is anticipated a 0.315D difference and standard deviation of 0.475D in the myopia progression between the intervention and control arms over a year. As an interim analysis will be performed at 6 months, the alpha level will be adjusted to 0.025 (2-tailed) based on Bonferroni adjustment. Considering a dropout rate of 10%, 65 participants will be needed in each arm to detect the difference with 90% power.

### Randomisation and masking

Subjects will be age-stratified into three subgroups: 4–6, 7–9, and 10–12 years, in a ratio of 1:2:1. Upon fulfilment of eligibility, they will be randomised to the experimental, control or auxiliary arm in an equal ratio. Minimization will be applied in the randomisation process to ensure balanced distribution of baseline myopia of subjects across the experimental and control arms. The randomisation list will be generated using a random number generator in an Excel spreadsheet. The group allocation will be performed by the trial manager. Unmasked investigators will receive the group assignment after randomisation, and they will be responsible for dispensing of spectacle lenses. Parents and subjects will not be informed about the group assignment during the study period. Masked investigators, without knowledge of the group assignments of subjects, will perform measurements for the primary and secondary outcomes at 6-month intervals. These investigators will not be allowed to touch or discuss the spectacle lenses of subjects during the eye examination.

### Study interventions

The experimental arm will be prescribed D2 spectacle lenses, which also named as DIMS Triple Enhanced Design. D2 is an experimental lens modified from the original DIMS spectacle lens in three ways: i) an increased magnitude of myopic defocus in each segment, ii) an increased number of segments, and iii) an expanded area of the treatment zone that contains segments. Both D2 and the original DIMS spectacle lens used polycarbonate as material. The control arm will be prescribed with single-vision spectacle lenses.

Participants will be asked to wear the study spectacles at all times, i.e., at least 8 hours per day and 7 days per week. Treatment compliance will be monitored at each follow-up visit through a questionnaire.

### Outcomes

The primary outcome is the change in cycloplegic SER over 12 months from baseline. The measurements will be performed by a masked investigator using an open-field autorefractor (Shin-Nippon NVision-K5001, Shin Nippon Yakugyo Co., Ltd., Tokyo, Japan). Cycloplegia is performed after applying 1 drop of topical anesthetic (Alcaine 0.5%), followed by 1 drop of cyclopentolate (Cyclogyl 1%) after 5 minutes. The full cycloplegic effect is confirmed when the amplitude of accommodation is reduced to 2D or less, 30 minutes after administering cycloplegia. If this is not achieved, another drop of cyclopentolate will be instilled. The first five readings with a variation of no more than 0.25D in both spherical and cylindrical components will be accepted and averaged.

The secondary outcome is the change in axial length over 12 months from baseline. The same masked investigator will be responsible for taking AL measurements after cycloplegia using a partial coherence interferometer (ZEISS IOLMaster 500, Carl Zeiss AG, Oberkochen, Germany). The first five readings with a variation of no more than 0.02 mm and signal-to-noise ratio above 3.5 will be accepted and averaged.

The choroid has been indicated to play a crucial role in the development of myopia in both animal and human studies [16,17]. Its thickness varies promptly to imposed retinal defocus. Chun et al found significant subfoveal choroidal thickening from 1 week to 2 years of DIMS spectacle lens wear [18]. Additionally, a predictive value of short-term changes of subfoveal choroidal thickness on long-term axial length changes is also indicated. In the current study, the choroidal thickness will be measured under un-dilated conditions using a swept-source Optical Coherence tomographer (Triton, Topcon Corporation, Tokyo, Japan). The correlation of changes of choroidal thickness with myopia control efficacy will be explored.

As DIMS spectacle lenses impose myopic defocus in peripheral retina, it is important to understand the peripheral optics of the retina and its influence on myopia development and control. Peripheral refraction will be performed using the same open-field autorefractor indicated for central refraction. Subjects will be fully dilated, and measurements will be taken at 10, 20, and 30 degrees nasally and temporally for the eligible eye.

**Fig 1:**
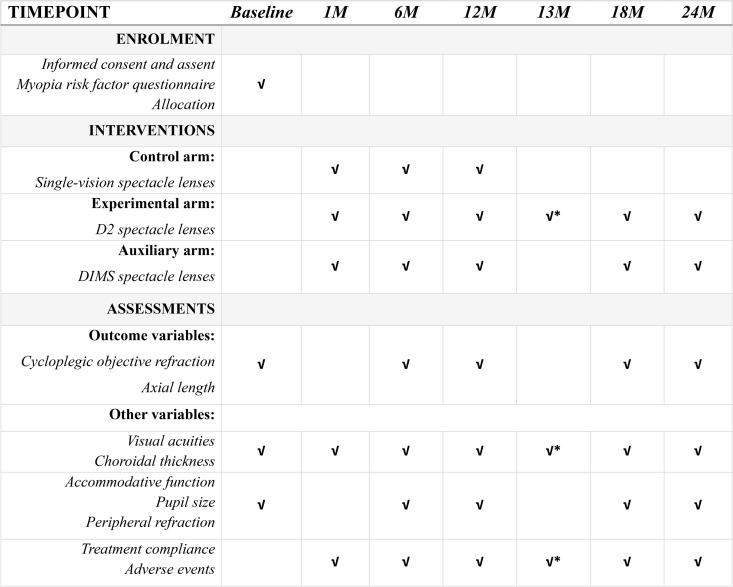
SPIRIT Schedule of enrolment, interventions, and assessments. Abbreviation: D2, new-generation Defocus Incorporated Multiple Segments; DIMS, original Defocus Incorporated Multiple Segments. *Only for subjects who crossover from the control arm to the experimental arm.

To monitor visual and ocular changes throughout the study period, distance best-corrected high contrast and low contrast visual acuity (by logMAR chart), intraocular pressure (by ICare tonometer or noncontact tonometer), accommodative amplitude (by near point ruler), accommodative lag (by near target and open-field autorefractor), pupil diameter in photopic condition (by pupillometer) and ocular health (by slit lamp biomicroscope and binocular indirect ophthalmoscopy) will also be evaluated. Genetic and environmental factors that may influence myopia progression must be considered. To address this, parents of participants are required to complete a validated myopia risk factor questionnaire at the baseline visit. A SPIRIT schedule of the study is presented in Fig 1.

### Procedures

Recruitment strategies include clinical record retrieval from the University Optometry Clinic databases, advertisements on social media platforms, and community vision screening. Preliminary determination of eligibility will be made through phone screening. Participants and parents who are interested and fulfil preliminary inclusion criteria will be scheduled for on-site screening. Written informed consent and assent will be obtained from parents and participants when eligibility is confirmed. Participants will be randomly allocated to one of the two arms after completion of the baseline visit. Spectacle lenses with a full cycloplegic subjective refraction will be made according to their group assignment. A 1-month follow-up will be scheduled to assess the vision and comfort of spectacle wear. Any adverse events will be recorded.

**Fig 2:**
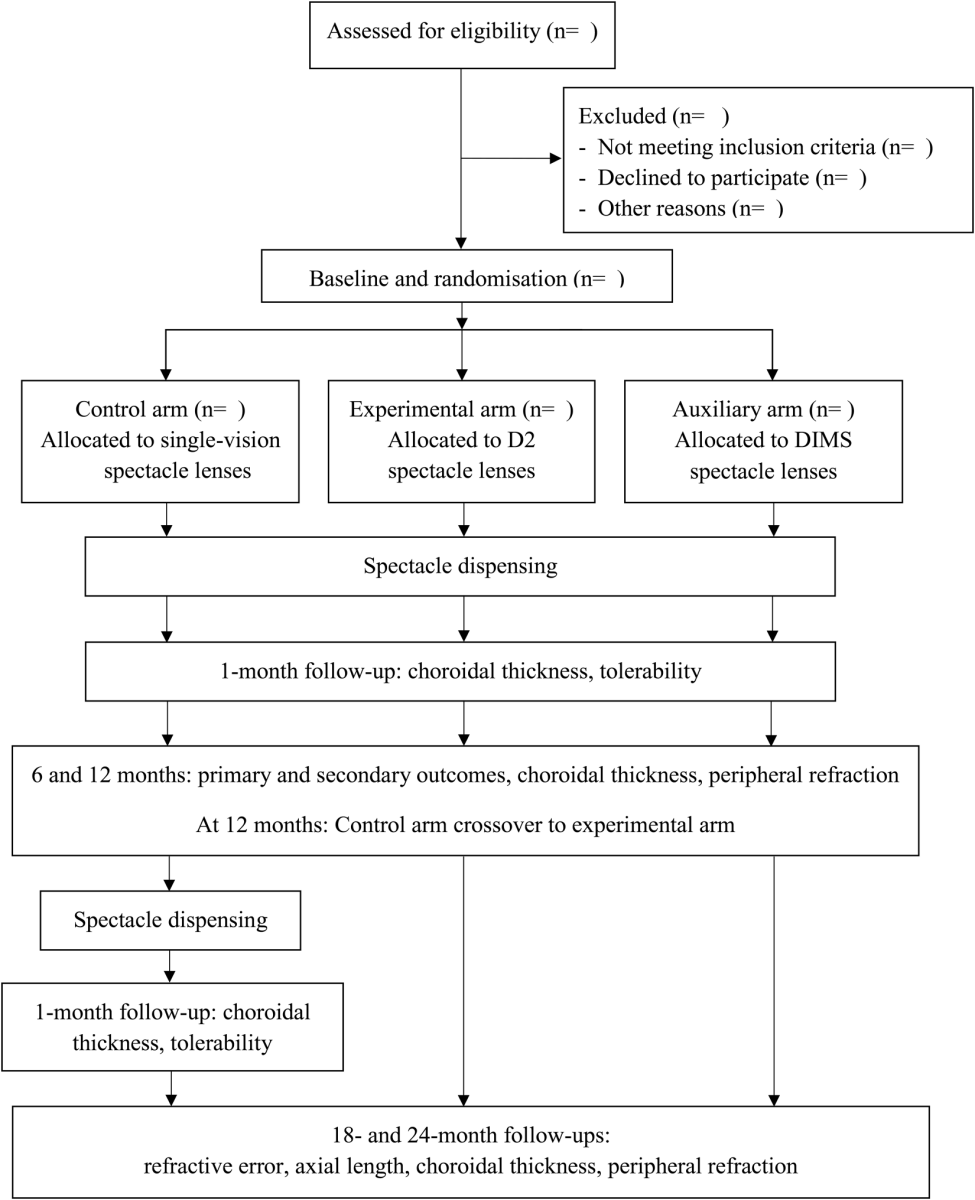
Study flow chart with timelines for the main outcomes. Abbreviation: D2, new-generation Defocus Incorporated Multiple Segments; DIMS, original Defocus Incorporated Multiple Segments.

Subjects will attend 6-monthly cycloplegic assessments at the Centre for Myopia Research during the 2-year study period. An update in prescription will be indicated if an increase or decrease of myopia by 0.50D or more, or the habitual visual acuity is equal to or worse than 0.20 logMAR, is found at any follow-up visit. After completion of the 1-year follow-up visit, subjects randomised to the control arm will crossover to the experimental arm and be provided with D2 spectacle lenses. To assess adaptation after crossover, an extra 1-month follow-up will be arranged. A study flow chart with timelines for the main outcomes is shown in Fig 2.

### Safety considerations

Previous RCTs have confirmed that DIMS spectacle lenses did not cause any adverse effects on visual function in myopic children [19]. Nevertheless, any adverse events, including reduction in visual acuity, visual disturbances, eye strain, headache, or nausea may or may not be causally related to the study intervention will be described, classified according to severity, and recorded. Any related serious adverse events will be reported to the IRB of PolyU within 48 hours.

### Data management plan

The source documents related to the study will include signed consent forms, printouts of ophthalmic investigations, and electronic case report forms (eCRFs). All paper documents will be stored in lockable cabinets. All study data will be recorded on standardized eCRFs and stored in an online database named Research Electronic Data Capture (REDCap) [20], in which the server is built and protected under a secured campus network. Access is granted only to authorized study investigators with unique account IDs and passwords, and only specific forms can be viewed and modified according to their designated roles. Anonymized subject identifiers will be used for any data entry and documents uploaded to REDCap. The Unmasked Investigators must complete each eCRF after all data have been entered. An independent monitor will review and endorse all the source data in each entry.

An independent data safety monitoring committee is not required for this myopia control trial due to its non-invasive nature [21]. Data safety and regular monitoring will be managed by the Principal Investigators and the trial manager.

### Statistical analysis

Interim and final analyses will be conducted by the Principal Investigators and the trial manager only. Data from the right eyes will be used for analyses when both eyes of the subjects satisfy the inclusion criteria. If only one eye is eligible, the specific eye will be used for analyses. The normality of the data will be examined using Kolmogorov-Smirnov tests. Demographic and optometric data will be displayed as mean and standard deviation, or number and percentage, as appropriate. The one-way analysis of variance and the Fisher’s exact test will be performed to compare baseline between-group differences in demographics (SER, age, sex).

The mixed repeated measures analysis of covariance will be used to test for significance in main outcomes and other continuous outcomes between arms, while controlling for covariates, including age and baseline myopia. The efficacy of myopia control of the DIMS spectacle lens will be determined by dividing the difference in myopia progression (or axial elongation) between the two arms by the myopia progression (or axial elongation) in the control arm, then multiplied by 100%. Multiple regression analysis will be employed to identify factors that may be associated with myopia control efficacy.

Data analysis will follow an intention-to-treat approach. Missing values of outcome variables and covariates will be replaced using multiple imputation procedures with 10 sets of imputations, assuming missing at random. All analyses will be 2-tailed with a significance level of 2.5%.

## Discussion

Studies from a variety of animal models have provided solid evidence that ocular growth and refractive development are actively controlled by optical defocus [22]. Specifically, wearing negative spectacles lenses on animal eyes imposed hyperopic defocus and stimulated eye growth towards myopia. In contrast, positive lenses produced myopic defocus, inhibited eye growth, and guided refractive development towards hyperopia. This compensatory eye growth was well controlled by the magnitude and sign of retinal defocus [23]. When animal eyes are exposed to simultaneous competing hyperopic and myopic defocus, the defocus signals may be averaged, or the myopic defocus may dominate refractive development, which leads to inhibition or even reversal of myopic eye growth [23–25]. This forms the scientific basis of current optical interventions for myopia control in children.

To date, two types of myopic defocus spectacle lenses with multiple segment design: the DIMS and the highly aspherical lenslets (HAL) spectacle lens have been clinically proven as safe and effective optical treatment modalities for controlling myopia progression in children [15]. Despite their distinctive optical and power distributions [26], the myopia control efficacy remains moderate [27]. Given parental concerns about the safety of contact lenses and pharmaceutical options, myopia control spectacle lenses are generally considered the first line of treatment strategy for young myopic children. Developing a new generation of myopia control spectacle lenses with higher efficacy offers significant scientific and clinical value.

This double-masked RCT is limited to one year, as control group subjects will receive treatment lenses in the second year. Given the availability of proven myopia control treatment options, it would be ethically controversial to withhold potentially beneficial treatments from control subjects for an extended period of time. The experimental and single-vision spectacle lenses are identical in appearance when viewed from the front. We speculate that a small number of scrupulous children or parents may be aware of the multiple segments from the specular reflection of light on the lens surface. Treatment compliance will be monitored by self-report questionnaires, making them susceptible to recall bias. The study is limited to participants of Chinese ethnicity, which may affect the generalizability of the findings to other populations.

## Conclusion

This study will offer valuable insights into the myopia control efficacy of a new generation of DIMS technology in children with early-onset and fast myopia progression. The findings can provide scientific evidence for eyecare practitioners to manage these high-risk cohorts.

## Dissemination plan

The Principal Investigators and the trial manager will have access to the trial dataset. The results of this study will be disseminated at scientific conferences and in peer-reviewed, indexed journals in optometry and ophthalmology. Upon publication of the study, parents and participants will be informed of the results, including their group allocation, through a newsletter.

## Supporting information

S1 File SPIRIT 2013 checklist. (DOCX)

S2 File Trial protocol. (PDF)
