# Supplementary material for: Myopia control efficacy of second-generation defocus incorporated multiple segments spectacle lenses on fast progressing myopes: Study protocol of a randomised controlled trial
Source: PLoS One. 2025 Oct 31;20(10):e0335061. doi: 10.1371/journal.pone.0335061 (PMC12578229; doi:10.1371/journal.pone.0335061)
Supplement: S2 File — (PDF) [file pone.0335061.s002.pdf]

Myopia Control Efficacy of Second-generation Defocus Incorporated Multiple Segments Spectacle Lenses on Fast Progressing Myopes: A Randomised Controlled Trial

PI: Dr. Dennis Yan-yin Tse

Co-PI: Prof. Carly Siu-yin Lam

School of Optometry, The Hong Kong Polytechnic University

Jointly funded by: HOYA Lens Thailand Ltd. and the Hong Kong Polytechnic University

Version number: v.6.0

(10 Jul 2025)

Summary of Changes from Previous Version 1.0:

Affected Section	Summary of Revisions Made	Rationale
Section 1	Short title has been revised	
Section 1 & 5.1	Inclusion criteria have been revised	For children aged 4-6 years old without past prescription forms, eligibility requires myopia below the 3rd percentile on refraction curves
Section 1 & 6.2	The selection of eye for statistical analysis has been revised	To provide a detailed description of the data selection for statistical analysis
Section 2	New visits, V5a & V5b, have been incorporated in the Study Plan Schematic, and conditions regarding lens changes have been [illegible]	To revise the Study Plan Schematic and provide a detailed description on conditions of lens changes
Section 5.1	A decision tree for selecting a valid past prescription form has been incorporated	To offer a clear guideline on the selection of eligible past prescription form
Section 5.11	- Clinical devices for measuring pupil size and IOP has been revised<br>- Measurement of BCVA has been updated<br>- Eyedrop for cycloplegia has been updated<br>- Measurement of peripheral refraction has been updated<br>- Measurement of ocular biometry has been deleted	To streamline data collection procedures and update measurement methods based on clinical needs
Section 5.14	Data collection time points for choroidal thickness and vision performance questionnaire have been updated	To clearly outline the data collection time points for each measurement
Section 5.15, 5.16, 5.17.2, 5.17.3	New visits, V5a & V5b, have been incorporated	To describe the study visits more accurately
Section 5.17	The record forms used in each study visit has been updated	To describe the record forms used more accurately
Section 5.18, 14.1	The record forms used in protocol violation have been updated	To describe the record form used more accurately
Section 1, 6.1 & 6.2	Sample size and statistical analysis have been revised	To introduce an interim analysis

Section 8.6	The procedures of data monitoring have been updated	More stringent monitoring has been implemented
Section 11.1	Disclosure of group allocation and thank-you letter to subjects after completion of study has been added.	To provide more detailed description of the actions upon study completion
Study Title	The full study title has been revised	To align the product name with that used in previous publications
Section 5.12 & 5.13	Timepoint for the main outcome measures has been revised	To correct the typographical error
Section 5.14	Timepoints for choroidal thickness measure has been revised	To introduce additional measurement timepoint to account for the lens change in control group
Study title	The full and short study titles have been revised by changing the word “Effectiveness” to “Efficacy”	“Efficacy” is more precise for a controlled clinical trial setting
/	Abbreviation of second-generation DIMS lens has been revised from “DG2” to “D2”	A simplified abbreviation is suggested by sponsor
Section 1	The funding source has been amended	To correct the errors in previous version
Section 1 & 6.1	Sample size calculation has been amended	To correct the errors in previous version

Table of Contents

1. Overview 9

2. Study Plan Schematic 15

3. Background and rationale of study 16

4. Study Objective 16

5. Study Design 17

5.1. Inclusion criteria 17

5.2. Exclusion criteria 19

5.3. Recruitment 19

5.4. Potential risks 20

5.5. Benefits 20

5.6. Study intervention 21

5.6.1. Phase 1: Two-arm RCT 21

5.6.2. Phase 2: Auxiliary group 21

5.7. Randomisation 21

5.8. Masking 22

5.9. Withdrawal criteria 22

5.10. Lost to follow-up 23

5.11. Baseline and eligibility assessment 24

5.12. Primary outcome measure 25

5.13. Secondary outcome measure 25

5.14. Other outcome measures 25

5.15. Study visits time windows 27

5.16. Assessments to be completed by visit 27

5.17. Data collection schedules 29

5.17.1. V1: Eligibility, randomisation and baseline assessment 29
5.17.2. V2 & V5a: Dispense 30
5.17.3. V3 & V5b: Adaption 30
5.17.4. V 4-7: Follow-up assessments (data related to treatment outcomes). 30
5.18. Protocol violations 30
6. Statistical Considerations 31
6.1. Sample Size Calculation 31
6.2. Statistical analysis 31
6.3. Data management 32
6.3.1. Source Data 32
6.3.2. Data recording and record keeping 33
7. Ethical approval and Consent 33
7.1. Human ethics approval 33
7.2. Informed consent/assent 33
8. Assessment of Safety/Adverse Event Reporting 34
8.1. Definitions 34
8.1.1. Adverse event (AE) 34
8.1.2. Serious Adverse Event (SAE) 34
8.1.3. Adverse device effect (ADE) 35
8.1.4. Serious adverse device effect (SADE) 35
8.1.5. Unanticipated problem (UP) 36
8.1.6. Device deficiency 36
8.2. Classification of an adverse event and adverse device effect 36
8.2.1. Causality 36
8.2.2. Severity 37

8.3. Responsibility 37

8.4. Reporting 37

8.5. Documentation and follow-up 38

8.6. Unmaking 38

8.7. Data safety and monitoring 39

9. Intervention supplies 39

9.1. Study treatment identification 39

9.2. Handling and dispensing of study treatment 40

9.3. Lens verification, packaging and labelling 40

9.4. Treatment supply records 40

10. Study completion 40

11. Dissemination of results 41

11.1. Study subjects 41

11.2. Academic/professional colleagues 41

12. Administrative Section 41

12.1. Adherence to the protocol 41

12.2. Protocol revision procedures 41

12.3. Case report form procedures 42

12.4. Monitoring/Source document verification 42

12.5. Data confidentiality and security 43

12.6. Reporting schedule 43

12.7. Record retention policy 43

12.8. Record Disposal 44

12.9. Insurance 44

12.10. Dissemination/Publicity Method 44

13. Abbreviations 44

14. Reference 47

Study Title:

Myopia Control Efficacy of Second-generation Defocus Incorporated Multiple Segments Spectacle Lenses on Fast Progressing Myopes: A Randomised Controlled Trial

Short Title:

Efficacy of DIMS on Fast Progressing Myopes

Principal Investigator:

Dr. Dennis Yan-yin Tse, Associate Professor, School of Optometry, The Hong Kong Polytechnic University (PolyU)

Co-Principal Investigator:

Prof. Carly Siu-yin Lam, Professor, School of Optometry, PolyU

Co-Investigators:

Prof. Chi-ho To, Chair Professor, School of Optometry, PolyU

Dr. Rachel Ka-man Chun, Research Assistant Professor, School of Optometry, PolyU

Dr. Jeffrey Tsz-wing Leung, Research Assistant Professor, School of Optometry, PolyU

Dr. Shanica Ying Hon, Optometrist, School of Optometry, PolyU

Ms Daisy Ka-yan Leung, Optometrist, School of Optometry, PolyU

Study Centre:

Optometry Research Clinic, School of Optometry, The Hong Kong Polytechnic University (Address: A137, 11 Yuk Choi Rd, Hung Hom, Hong Kong)

Anticipated commencement date: Aug 2023

Anticipated end date: Sep 2026

1. Overview

Title of study

Myopia Control Efficacy of Second-generation Defocus Incorporated Multiple Segments Spectacle Lenses on Fast Progressing Myopes: A Randomised Controlled Trial

Short title

Efficacy of DIMS on Fast Progressing Myopes

Study description and methodology

Myopia is a widespread concern of global significance; however, it remarkably impacted many East Asian populations, including Hong Kong. The prevalence of myopia in 8-year-old Hong Kong children has reached approximately 40%.[1,2] In most cases, myopia developed in early childhood will progress until late adolescence. As our population ages, it is projected that more than 80% of the population will become myopic, of whom 10% will be highly myopic.[3] Notably, high myopia is associated with the increasing risk of sight-threatening eye diseases, such as glaucoma, retinal detachment, choroidal neovascularization and myopic macular degeneration.[4-7] It can cause an increasingly high socio-economic burden to the health care system and society.[8, 9]

Various interventions have been implemented to halt myopia progression in children, yielding efficacy rates ranging from 15% to 60%.[10] The clinical application of myopic defocus in conjunction with spectacle correction, through the Defocus Incorporated Multiple Segments (DIMS) technology, has demonstrated to be a viable and effective method for slowing down the progression of myopia in children.[11] However, DIMS spectacle lenses (first generation, D1) reduced myopia progression by 52% and axial elongation by 62% in children aged 8-13 years[11] may not convincingly reflect the technology's effectiveness on fast progressing myopes and younger myopes. PolyU and spectacle lens manufacturer (HOYA Corporation, Tokyo, Japan) jointly designed

the second generation of DIMS lens (D2) that aims to provide a higher effectiveness than the first-generation lens.

The aim of this Randomised Clinical Trial (RCT) is to validate the efficacy of the first and second generation of DIMS lenses (D1 & D2) to reduce myopia progression for fast progressing myopic children aged 4-12 years. To investigate factors affecting myopia control efficacy between individuals, relative peripheral refraction (RPR) and choroidal thickness measurements are also conducted.

Objective

Primary:

i. To determine the efficacy of D2 lens design in slowing myopia progression in children with fast progressing myopia as compared to the single vision (SV) control.

Secondary:

i. To determine the efficacy of D1 lens design in slowing myopia progression in children with fast progressing myopia as compared to SV control.
ii. To investigate if baseline RPR influences the outcome of myopia control when using DIMS lenses.
iii. To determine if the change of choroidal thickness following short-term DIMS lens wear has a prognostic value in determining the efficacy of myopia control over one year.

Study population

One hundred and seventy-seven fast progressing myopic children between the ages of 4 to 12 years will be recruited. They will be stratified by 3 age groups: 4-6, 7-9 and 10-12, in a ratio of 1:2:1.

Sites of the study

Optometry Research Clinic, School of Optometry, PolyU

Inclusion criteria

- Hong Kong Chinese
- Age at enrolment: 4-12 years
- Myopia (in spherical equivalent refraction, SER) -0.75D or below in both eyes
- Documented evidence of fast progressing myopia in the last 2 years, either in SER or axial length (AL) (Details in Section 5.1):
  - SER progression: 0.50D/year or more in either one eye or both eyes
  - AL elongation: 0.27mm/year or more in either one eye or both eyes
- For children aged 4-6 years without past prescription forms, eligibility requires myopia below the 3rd percentile on refraction curves:[12]
  - 4-5 years old: myopia of -0.75D or below in both eyes
  - 6 years old: myopia of -1.25D or below in at least one eye, with the other eye -0.75D or below
- Best-corrected visual acuity (BCVA) in both eyes matches age norms: [13, 14]
  - 4-6 years old: 0.20 logMAR (or its equivalent) or better
  - 7-12 years old: 0.00 logMAR (or its equivalent) or better
- Anisometropia of 1.50 D or less.
- Astigmatism of 2.00 D or less.
- Acceptance of random group allocation and the masked study design
- Able to wear the prescribed spectacle full-time

Exclusion criteria

- Existing or past eye diseases or surgeries (e.g., strabismus surgery, amblyopia, oculomotor nerve palsies, corneal disease, intraocular disease, etc.) that may have an impact on vision or visual development
- Binocular vision problems
- Long-term medication (intake at least 3 days/week)
- Medication or supplements that affect eye growth
- Systemic diseases that may have an impact on vision or visual development (e.g., endocrine, cardiac and respiratory diseases, diabetes, Down syndrome, etc.)
- Previous or current treatment for myopia control (e.g., orthokeratology, progressive addition lenses, myopic defocus lenses, atropine, etc)

- Allergies to topical anaesthetic or cyclopentolate eye drops
- Individuals who, in the judgment of the Investigator, are unable to cooperate and follow instructions during eye examination.

Randomisation

After baseline measurements, subjects will be Randomised to three groups at a ratio of 1:1 using a stratified randomisation based on age. Age will be stratified into 3 groups: 4-6, 7-9, and 10-12, in a ratio of 1:2:1.

Criteria for evaluation

Primary efficacy outcome

- Change in SER with cycloplegic auto-refraction over 12 months

Secondary efficacy outcomes

- Change in AL over 12 months

Other outcomes

- Change in relative peripheral refraction
- Change in choroidal thickness
- Change in accommodative status
- Treatment compliance: lens wear duration per day and number of days per week

Other optometric outcomes not listed here will be considered exploratory in nature.

Primary safety outcome

- Adverse events, including biomicroscopic findings, for the treatment and control groups over 2 years

Statistical methods

Sample Size Calculation

To achieve a 90% power to detect a 0.315D difference[11] and standard deviation of 0.475D (effect size of 0.658) in SER progression between a treatment group and a control group with an alpha level of 0.05 (2-tailed); the minimum subject number required in each group is 50.

An interim analysis will be performed to evaluate the efficacy on primary and second outcomes at 6 months. To compensate the impact on the final efficacy analysis, Bonferroni adjustment will be performed and the adjusted alpha level will be 0.025. Accordingly, the subject number required in each group is 59 at a 90% power. Assuming a dropout rate of about 10%, at least 65 subjects are required in each group.

Statistical analysis

Data from the right eye will be used for analyses when both eyes show fast progression. If only one eye demonstrated fast progression, that specific eye would be used for analyses. Demographic and optometric data will be displayed as mean and standard deviation, or number and percentage as appropriate. The Chi-square test (or, if appropriate, Fisher's Exact test) and unpaired t-test will be performed to compare baseline between-group differences in demographics (age, sex). Myopia progression will be calculated as the difference between SER and AL at the baseline, the 6-month and the 1-year visits, respectively.

The unpaired t-test will be used to compare the changes in primary and secondary outcomes between groups. The efficacy of myopia control of DIMS lens will be determined by dividing the difference in myopia progression (or axial elongation) between two groups with the myopia progression (or axial elongation) in the SV group, then multiplied by 100%. Multiple regression analysis will be employed to identify factors that may associated with myopia control efficacy.

Data analysis will follow an intention-to-treat approach. Missing values of outcome variables and covariates will be replaced using multiple imputation procedures with 10 sets of imputations assuming missing at random. All analyses will be 2-tailed with a significance level of 2.5%.

The CONSORT Statement will guide the reporting of results.

Funding

The study is jointly funded by The Hong Kong Polytechnic University and Hoya Lens Thailand Ltd. The D1 and D2 spectacle lenses are developed based on DIMS technology in collaboration with Hoya Lens Thailand Ltd.

2. Study Plan Schematic

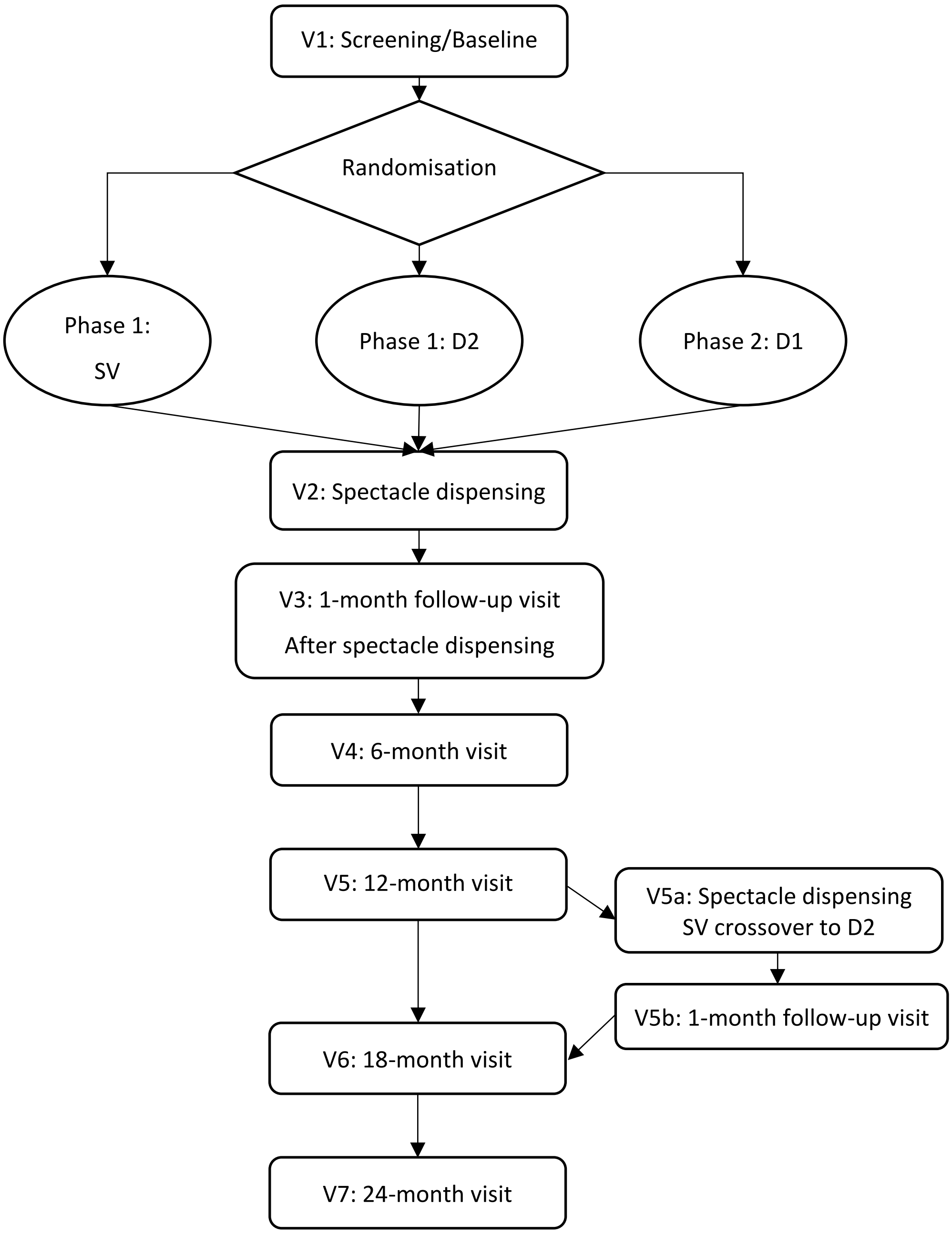

Remarks: At each 6-month visit, the criteria for updating the prescription of spectacle lenses for all groups include any of the following:

- Increase in myopia of 0.50D (spherical over-refraction) or more, in either one eye or both eyes
- Habitual visual acuity equal to or worse than 6/9.5, in either one eye or both eyes

An additional spectacle dispensing visit will be scheduled after a prescription update.

3. Background and rationale of study

The significant worldwide increase in myopia has ignited increased research interest in predicting and halting myopia development. Myopic defocus has been shown to slow axial growth and myopia progression, primarily based on the principle of simultaneous competing defocus and peripheral optics. Orthokeratology, concentric ring soft contact lenses and DIMS spectacle lens are clinical applications that utilize myopic defocus for slowing down myopia progression in children. However, none of the above was fully effective (~40-60%) in controlling myopia progression.

DIMS technology is developed by PolyU and HOYA. In the 2-year RCT conducted \between 2014-2017, DIMS spectacle lens significantly reduced myopia progression by 52% and axial elongation by 62% in schoolchildren aged 8-13 years. Subsequently, it has been marketed globally under the trade name of MiyoSmart, achieving commercial success due to its non-invasiveness and cost-effectiveness from the customers' perspective. Recently, the joint research team has designed the second generation of DIMS lens (D2) which aims to provide a higher effectiveness compared to the previous design. Despite their higher risk of progressing to higher levels of myopia, limited attention has been given to fast progressing myopes and younger myopes. To explore and enhance the effectiveness of myopia control in these specific groups, both D1 and D2 lenses will be applied as investigational treatments in the current RCT.

4. Study Objective

Primary:

i. To determine the efficacy of D2 lens design in slowing myopia progression in children with fast progressing myopia as compared to SV control.

Secondary:

ii. To determine the efficacy of D1 lens design in slowing myopia progression in children with fast progressing myopia as compared to SV control.
iii. To investigate if baseline RPR influences the outcome of myopia control when using DIMS lenses.

iv. To determine if change of choroidal thickness following short-term DIMS lens wear has a prognostic value in determining the efficacy of myopia control over one year.

5. Study Design

5.1. Inclusion criteria

The study will enroll participants aged between 4 and 12 years and of Hong Kong Chinese descent. Both eyes of the subjects should have a SER of -0.75D or lower. Except for children aged 4-6 years, documented evidence of fast progressing myopia over the past 2 years, either in terms of SER or AL, is required for enrollment. Documented evidence is defined as past prescription (Rx) forms issued by registered optometrist or registered ophthalmologist within 2 years before the baseline date. Fast progression is defined as a SER progression at a rate of 0.50D/year or more, or an AL elongation at a rate of 0.27mm/year or more in either or both eyes. Documented evidence is selected using the decision tree outlined below.

For children aged 4-6 years without past Rx forms, eligibility requires myopia below the 3rd percentile on refraction curves. Specifically, for 4 to 5-year-olds, their myopia should be -0.75D or below in both eyes. For 6-year-olds, their myopia should be -1.25D or below in at least one eye, with the other eye at -0.75D or below.

BCVA in both eyes should match age norms. For children aged 4-6 years, BCVA of 0.20 logMAR (or its equivalent) or better is necessary, whereas children aged 7-12 years should have BCVA of 0.00 logMAR (or its equivalent) or better. Anisometropia should not exceed 1.50D, and astigmatism should be equal to or less than 2.00D. Participants must demonstrate a willingness to accept random group allocation and adhere to the masked study design. Moreover, they should be able to wear the prescribed spectacles full-time.

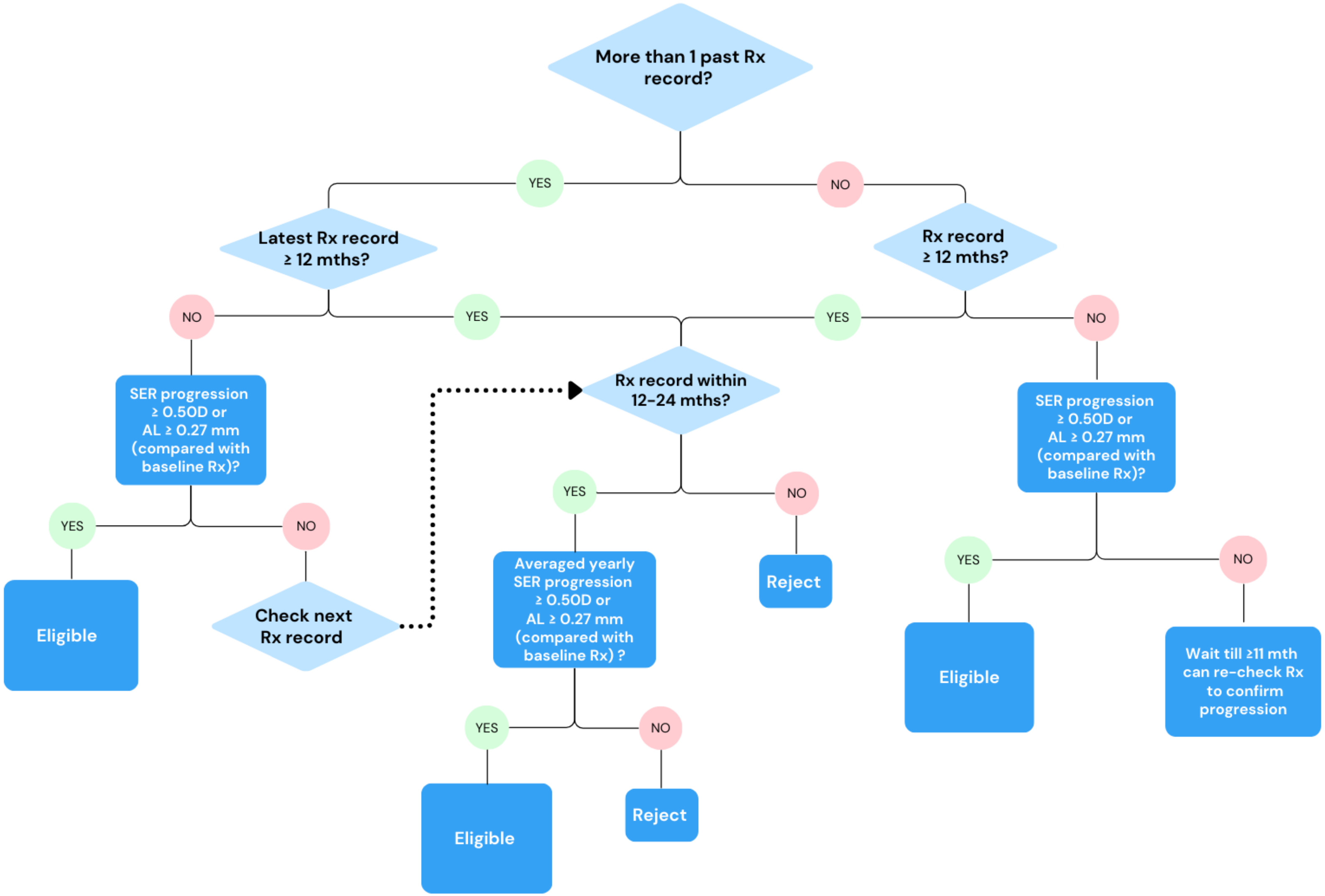
More than 1 past Rx record?
YES
NO
Latest Rx record ≥ 12 mths?
Rx record ≥ 12 mths?
NO
YES
YES
NO
SER progression ≥ 0.50D or AL ≥ 0.27 mm (compared with baseline Rx)?
Rx record within 12-24 mths?
SER progression ≥ 0.50D or AL ≥ 0.27 mm (compared with baseline Rx)?
YES
NO
YES
NO
Eligible
Check next Rx record
Averaged yearly SER progression ≥ 0.50D or AL ≥ 0.27 mm (compared with baseline Rx) ?
Reject
YES
NO
Eligible
Wait till ≥11 mth can re-check Rx to confirm progression
YES
NO
Eligible
Reject

5.2. Exclusion criteria

An individual who meets any of the following criteria will be excluded from participation in this study: individuals with any existing or past eye diseases or surgeries, including but not limited to strabismus surgery, amblyopia, oculomotor nerve palsies, or corneal disease, intraocular disease, or any other conditions that may have an impact on vision or visual development, are excluded; those with binocular vision problems are also excluded. Individuals who regularly intake long-term medication for at least three days per week or use medications or supplements known to affect eye growth are ineligible for the study. Additionally, individuals with systemic diseases that may impact vision or vision development, such as endocrine, cardiac and respiratory diseases, diabetes, Down syndrome, or similar conditions, are excluded. Previous or current treatment for myopia control, such as orthokeratology, progressive addition lenses, myopic defocus lenses, or atropine, is considered as an exclusion criterion. Individuals with allergies to topical anesthetic or cyclopentolate eye drops are not eligible to participate. Finally, individuals who, in the judgement of the Investigator, are unable to cooperate or follow instructions during eye examinations are excluded from the study.

5.3. Recruitment

Strategies for subject recruitment include:

1. PolyU optometry clinic and research clinic databases: Identify potential participants through a search of clinic records. Initial contact will be made via telephone. Information regarding study's objectives, procedures, potential risks, and benefits will be verbally introduced to the parent/guardian.
2. Collaborate with schools: Establish partnerships with local schools by offering eye health talks to parents and children. During these talks, information regarding the research study will be introduced. Any concerns or questions from parents can be addressed immediately at the same session. Request permission to include subject recruitment poster in school newsletters or circulars to reach parents.
3. Community outreach: Collaborate with district councils or community service organization to organize eye health talks or vision screenings to raise awareness

about this research study. Any concerns or questions from parents and participants can be addressed immediately at the same session.

4. Online platforms: Prepare advertisements for subject recruitment in the form of a YouTube video and poster. Seek an advertising agency to leverage social media platforms, websites, or online forums popular among parents in Hong Kong to spread the advertisements and reach a larger audience.
5. Parental influence: Invite parents of current or previous subjects from our recent research studies to spread the advertisement for subject recruitment to other parents groups.

5.4. Potential risks

The eye examination should not result in any undue discomfort. A mild stinging sensation may be experienced for a few seconds when the eye drops for refraction are instilled. The subject may also experience blurry near vision and sensitivity to bright light for about 24 hours after the instillation of eye drops. Therefore, it is suggested to wear sunglasses or a hat while going outdoors. An information sheet regarding the eye drops used in the study will be provided to participants.

The treatment interventions, specifically the spectacle, may induce blurriness, dizziness, and interrupted binocular vision at the first few days of lens wear. A one to two weeks' adaptation period may be required until the symptoms resolved. Upon experiencing persistent discomfort while wearing the spectacle, subjects or parents should report to the Investigators via phone call or message (Dr. Shanica Hon or Ms. Daisy Leung, Tel: 6473 5750).

5.5. Benefits

The refractive status and eye health conditions of the participants will be closely monitored by eye care professionals throughout the 2-year study period. The treatment interventions may slow down the myopia progression of the participants. Participants and parents do not need to pay for eye examinations or spectacles.

This research study aims to demonstrate the safety and efficacy of new spectacle lenses in reducing myopia progression in children. A positive outcome is likely to accelerate the global commercialization of this clinical management option, thereby reducing the enormous socioeconomic burden associated with vision loss due to myopia and high myopia.

To express gratitude to the participants and parents for their valuable time and contribution to the research study, they will receive either a pair of single vision spectacles or MiyoSmart spectacles upon completion of the study.

5.6. Study intervention

5.6.1. Phase 1: Two-arm RCT

This study is a double-masked RCT. Subjects and parents are masked from the type of investigational lenses they are wearing. Masked Investigators will be responsible for masked measurements of the primary and secondary efficacy outcomes at the baseline and 6-monthly visits. Masked Investigators will never examine the subjects while they are wearing their spectacle lenses. Unmasked Investigators will conduct the remaining measurements and dispense the investigational lenses.

Eligible subjects will be randomly assigned to the control or the treatment group in an equal ratio. The control group will receive placebo SV lenses, while the treatment group will receive D2 lenses.

Scheduled visits will be conducted over a period of 2 years. The control group will cross over to wear D2 lenses at 12 months and all subjects will continue wearing D2 lenses for an additional 12 months.

5.6.2. Phase 2: Auxiliary group

To compare the efficacy and visual performance of D1 and D2 lenses for internal reference, an auxiliary group having the same number of subjects will be assigned to wear D1 lenses for 2 years. Recruitment criteria, measurement and follow-up intervals of the auxiliary group will be the same as in Phase 1 study.

5.7. Randomisation

Simple randomisation will be implemented by an unmasked Investigator by creating a column of random numbers for the group allocation using Microsoft Excel. Eligible subjects will be assigned to one of the three groups in an equal ratio by following the random sequence generated from Excel. Age will be stratified by 3 groups: 4-6, 7-9 and 10-12, in a ratio of 1:2:1.

5.8. Masking

The study will be double-masked. The primary and secondary outcome assessments will be performed by masked Investigators and the subject and parents/guardians will be masked to group allocation and study intervention until the data analysis is completed.

Masked Investigators will perform masked measurements of the primary and secondary efficacy outcomes (cycloplegic auto-refraction and axial length) at every 6-month visit. The masked Investigators will not be allowed to enter subjects' eye examination room and check their glasses, other than to perform their masked examination duties. Subjects will be told not to discuss their lens wear with the masked Investigator.

The children will be asked to wear the spectacles full-time. Compliance will be regularly monitored at every follow-up visit and through the completion of a questionnaire regarding their lens use.

5.9. Withdrawal criteria

Subjects (or parents/guardians for subjects <18 years of age) are free to withdraw from participation in the study at any time upon request. No further data will be collected on subjects who withdraw from this study.

If significant intolerance to the treatment intervention is suspected at any time after randomisation, subjects can choose to withdraw or terminate treatment (either treatment or control group).

An Investigator may discontinue or withdraw a subject from the study for the following reasons:

- Significant study intervention noncompliance: unable to contact via 3 phone calls and 3 messages, more than 3 attempts to arrange missed follow-up appointments, wearing the spectacles less than 5 days a week and/or an average of less than 5 hours/day.
- If any clinical adverse event or situation occurs (e.g., allergies to eye drops, reduction of visual function such as diplopia, reduction of BCVA; subject to clinicians' judgement) such that continued participation in the study would not be in the best interest of the subject.

- If the participant meets an exclusion criterion (either newly developed or not previously recognized) that precludes further study participation.

Should subjects require discontinuation of treatment intervention for any reason, follow-up visits and data collection will not continue as scheduled. The reason for discontinuation or withdrawal from the study will be recorded on the Discontinuation/Withdrawal Case Report Form (CRF) in a web-based secure remote electronic database named Research Electronic Data Capture (REDCap). Subjects who signed the informed consent form and were Randomised but did not receive treatment intervention may be replaced. If there are subjects who signed the informed consent form, were Randomised, received the study intervention but were discontinued or withdrawn within the first month due to not meeting inclusion criteria (e.g., unable to tolerate spectacles or eye drops), and it is still within the recruitment period, then these participants' randomisation will be discarded and replaced with a participant from the next allocation. All spectacles (used or unused) will be returned to the unmasked Investigator upon withdrawal or discontinuation from the study.

5.10. Lost to follow-up

A subject will be considered lost to follow-up if they fail to return for at least one of the data collection visits and is unable to be contacted by study staff. The following actions must be taken if a subject fails to return to the research clinic for a required study visit:

- The Investigators will attempt to contact the subject and reschedule the missed visit within 2 weeks of the original schedule and counsel the subject on the importance of maintaining the assigned visit schedule and ascertain if the subject and their family wishes to and/or should continue in the study.
- Before a subject is deemed lost to follow-up, the Investigator will make every effort to regain contact with the participant (where possible, 3 telephone calls and 3 messages, if necessary, a letter to be sent to the participant's last known mailing address or email). These contact attempts will be documented in the subjects' study file.
- Should the subject continue to be unreachable, they will be considered to have withdrawn from the study with a primary reason of lost to follow-up.

5.11. Baseline and eligibility assessment

All data will be collected via face-to-face meetings with Investigators at the Optometry Research Clinic of School of Optometry in PolyU. The following data will be collected at the baseline and eligibility assessment according to the manual of procedures.

- **Demographic data:** ethnicity, age (date of birth) and gender.
- **Concomitant medication:** information about types and dose of medications or supplements currently used (will be asked at all visits).
- **Allergies history:** information about any allergies to medication or others (will be asked at all visits).
- **Questionnaire:** information about the participants' visual habits and parental history of myopia.
- **Presence of strabismus:** measured using the cover test at distance and near.
- **Autorefraction and keratometry:** measured using a Shin-Nippon NVision-K 5001 open-field autorefractor.
- **Pupil size:** measured using VIP-300 Pupillometer under photopic condition.
- **Subjective refraction:** measured using trial frame and loose lenses (most plus before blur), Jackson cross cylinder for astigmatism and Thomson Test Chart 2000 system.
- **Objective refraction:** measured using retinoscopy, trial frame and loose lenses and Thomson Test Chart 2000 system.
- **BCVA:** High contrast BCVA measured monocularly and binocularly at distance and near using the logarithmic visual acuity charts "ETDRS". Low contrast BCVA measured binocularly at distance using the 10% Michelson low contrast logarithmic visual acuity charts "ETDRS".
- **Binocular vision with dry subjective refraction:** stereopsis measured using a Randot test, monocular and binocular amplitude of accommodation measured using a RAF ruler, monocular accommodative lag measured with a 3D stimulus using a Shin-Nippon NVision-K 5001 open-field autorefractor, and phoria measured using a Howell Phoria Card at distance and near.
- **Intraocular pressure (IOP):** IOP will be measured by Topcon CT-80 non-contact tonometer or ICare tonometer.
- **External ocular health examination:** using a slit lamp biomicroscope.

- **Cycloplegia:** One drop of Alcaine 0.5% or Provain 0.5% (topical anaesthetic) and 1 drop of Cyclopentolate hydrochloride 1.0% (antimuscarinic eye drop) will be instilled to induce cycloplegia. The effect of the cycloplegia will be evaluated by means of amplitude of accommodation (push-up method); if the amplitude of accommodation is more than +2.00D, then a second drop of 1% Cyclopentolate will be instilled. The remaining tests of this section will be performed under cycloplegia.
- **Cycloplegic autorefraction:** same procedure as the non-cycloplegic autorefraction.
- **Peripheral refraction:** measured by using a Shin-Nippon NVision-K 5001 autorefractor at center, 10°, 20° and 30° of the nasal and temporal visual field across the horizontal meridian in the right eye (if both eyes confirm fast progress) or in the fast-progress eye (if only 1 eye confirmed fast progress). Five measurements will be taken and averaged.
- **Cycloplegic subjective refraction:** same procedure as the non-cycloplegic subjective refraction.
- **Axial length:** measured by ZEISS IOLMaster 500.
- **Dilated fundus ophthalmoscopy:** posterior eye health will be checked using a binocular indirect ophthalmoscopy and fundus photo camera.

5.12. Primary outcome measure

The primary outcome will be the change in SER from baseline to 12 months, as measured by cycloplegic autorefraction (Shin-Nippon open-field autorefractor) in both eyes.

5.13. Secondary outcome measure

The secondary outcome measure is the change in AL under cycloplegia from baseline to 12 months.

5.14. Other outcome measures

- **Choroidal thickness:** measured using a Topcon DRI OCT Triton optical coherence tomography at spectacle dispensing, 1 month after spectacle dispensing, 6, 12, 13 (for control group crossover to D2 group), 18, 24 months.
- **Corneal curvature:** measured at 6, 12, 18, 24 months.

- **Peripheral refraction:** at centre, 10˚, 20˚ and 30˚ of the nasal and temporal visual field across the horizontal meridian in right eye at 6, 12, 18 and 24 months.
- **Heterophoria:** measured at 6, 12, 18, 24 months.
- **Pupil size:** measured in normal lighting conditions at 6, 12, 18, 24 months.
- **Amplitude of accommodation (binocular and monocular, with trial frame)**: measured at 6, 12, 18, 24 months.
- **Accommodative lag (with trial frame):** measured with a 3D stimulus at 6, 12, 18, 24 months.
- **IOP:** measured at 6, 12, 18, 24 months
- **Visual performance questionnaire:** measured at 1 month after spectacle dispensing and 1 month after control group crossover to D2 group.
- **Treatment compliance:** spectacle lens wear duration per day and number of days per week monitored at 1 month after spectacle dispensing, 6, 12, 18, 24 months.
- **Adverse events and device events:** Information regarding to any adverse events and whether they are related to the active treatment will be collected at all the time points.

All other measurements will be classified as exploratory outcomes.

5.15. Study visits time windows

Visit Number	Target Visit Window (days)	Acceptable Visit Window (days)
V1 (Baseline)	0	0
V2 (dispensing)	30	15-45
V3 (1-month after dispensing)	60	53-67
V4 (6-month)	180	150-210
V5 (12-month)	360	330-390
V5a (dispensing)*	390	375-405
V5b (1-month after dispensing)*	420	413-427
V6 (18-month)	540	510-570
V7 (24-month)	720	690-750

* Only applicable for control group

5.16. Assessments to be completed by visit

Standard procedures for each assessment are listed in the Manual of Procedures.

Assessment	Phone Screening	Screening	V1: Baseline	V2 & V5a: Spectacle dispensing (On indication after V3-6)	V3 & V5b: 1-month after spectacles dispensing	V4-6: 6-monthly	V7: 24-month
Informed consent/assent			X				
Demographics	X	X					
Medical history	X	X	X	X	X	X	X
Refractive status history (last 2 years)	X	X					
Myopia risk factor questionnaire			X				
Randomisation			X				
Administer study intervention				X		(X)	
VA by ETDRS (habitual/unaided; Distance & Near) (>6 yo)			X		X	X	X
VA by HOTVUX (habitual/unaided; Distance & Near) (4-6yo)			X		X	X	X

Cover test		X					
Colour vision		X					
Interpupillary distance			X			X	X
Non-cycloplegic autorefraction and keratometry			X		X	X	X
Non-cycloplegic subjective refraction (>6 yo)			X			X	X
Non-cycloplegic objective refraction by retinoscopy (4-6yo)			X			X	X
BCVA by ETDRS (Distance & Near) (>6 yo)			X			X	X
BCVA by HOTVUX (Distance & Near) (4-6yo)			X			X	X
BCVA by low contrast ETDRS OU (>6 yo)			X				
BCVA by low contrast HOTVUX OU (4-6yo)			X				
VA with new spectacles by ETDRS (>6 yo)				X			
VA with new spectacles by HOTVUX (4-6yo)				X			
VA with new spectacles by low contrast ETDRS OU (>6 yo)				X			
VA with new spectacles by low contrast HOTVUX OU (4-6yo)				X			
Phoria			X			X	X
Anterior ocular health		X	X			X	X
IOP			X			X	X
Pupil size			X			X	X
Stereopsis		X	X				
Amplitude of accommodation			X			X	X
Accommodative lag			X			X	X
OCT				X	X	X	X

Cycloplegia			X			X	X
Cycloplegic autorefraction			X			X	X
Peripheral refraction			X			X	X
Cycloplegic subjective refraction (>6 yo)			X			X	X
Cycloplegic objective refraction by retinoscopy (4-6yo)			X			X	X
Axial length			X			X	X
Internal ocular health (central & periphery)			X				X
Fundus photo			X				X
Order spectacle lenses			X			(X)	(X)
Verification of spectacle lenses				X	X	X	X
Frame adjustment				X	(X)	(X)	(X)
Spectacle lenses dispense				X			
Visual performance questionnaire					X		
Treatment compliance					X	X	X
Adverse event review and evaluation			X	X	X	X	X
Complete CRF		X	X	X	X	X	X

5.17. Data collection schedules

The following steps will be conducted by study Investigators:

5.17.1. <u>V1: Eligibility, randomisation and baseline assessment</u>

1. Screen potential participants over the telephone (recorded in electronic spreadsheet)
2. Enter contact details, demographics, and record refractive history (recorded in electronic spreadsheet)
3. Perform baseline assessment on participants for eligibility (recorded in REDCap CRF_V1)
4. Randomisation of participant (recorded in electronic spreadsheet)

5.17.2. V2 & V5a: Dispense

1. Order the type of spectacle lenses (SV/D1/D2 lenses) after V1 according to randomisation
2. Dispense treatment spectacles (recorded in REDCap CRF_V2 & REDCap CRF_V5a)

5.17.3. V3 & V5b: Adaption

1. Measure visual performance (recorded in Qualtrics Survey)
2. Record treatment compliance (recorded in REDCap CRF_V3 & REDCap CRF_V5b)
3. Perform choroidal thickness measurement

5.17.4. V 4-7: Follow-up assessments (data related to treatment outcomes)

The following steps will be completed by study Investigators at the 6, 12, 18 and 24-month follow-up visits:

1. Record results of follow-up visual assessments (recorded in REDCap CRF_V4-7)
2. Record treatment compliance (recorded in REDCap CRF_ V4-7)
3. Record any adverse events affecting the participant and any incidental events (recorded in REDCap CRF_AE)
4. Update spectacle lenses if required (recorded in REDCap CRF_V2 & V5a)

5.18. Protocol violations

A deviation from the protocol when no amendment has been submitted and approved would be regarded as a protocol violation. All protocol violations must be documented and reported (recorded in electronic spreadsheets). The prescribed treatment dose is at least 10 hours of spectacle wear per day, 7 days a week (i.e., at least 70 hours each week). A subject is considered as compliant with the study protocol if he/she has worn the spectacles for at least 56 hours a week (7 days of at least 8 hours each). If the spectacle lenses are worn for less than 35 hours per week or for less than 3 days per week, it is defined as a protocol violation. A list of such subjects will be prepared prior to unmasking.

6. Statistical Considerations

6.1. Sample Size Calculation

To achieve a 90% power to detect a 0.315D difference[11] and standard deviation of 0.475D (effect size of 0.658) in SER progression between a treatment group and a control group with an alpha level of 0.05 (2-tailed); the minimum subject number required in each group was 50.

An interim analysis will be performed to evaluate the efficacy on primary and second outcomes at 6 months. To compensate for the impact on the final efficacy analysis, Bonferroni adjustment will be performed with an adjusted alpha level of 0.025. Accordingly, the subject number required in each group is 59 at a 90% power. Assuming a dropout rate of about 10%, at least 65 subjects are required in each group.

6.2. Statistical analysis

Data from the right eyes will be used for analyses when both eyes show fast progression. If only one eye desmonstrated fast progression, that specific eye would be used for analyses. Demographic and optometric data will be displayed as mean and standard deviation, or number and percentage as appropriate. The Chi-square test (or if appropriate, Fisher's Exact test) and unpaired t-test will be performed to compare baseline between-group differences in demographics (age, sex). Myopia progression will be calculated as the difference between SER and AL at the baseline, the 6-month and the 1-year visits, respectively.

The unpaired t-test will be used to compare the changes in primary and secondary outcomes between groups. The efficacy of myopia control of DIMS lens will be determined by dividing the difference in myopia progression (or axial elongation) between two groups with the myopia progression (or axial elongation) in the SV group, then multiplied by 100%. Multiple regression analysis will be employed to identify factors that may associated with myopia control efficacy. Regarding visual performance and comfort, subjective ratings collected by questionnaires will be compared between groups using Mann-Whitney U test.

Treatment will be discontinued if any treatment-related adverse event arises, such as constant visual disturbance (e.g., ghosting, diplopia, glare, etc.). The number of subjects

discontinuing treatment prematurely for any reason will be summarised by reasons for discontinuation. The incidence of all serious adverse events will be summarised.

Data analysis will follow an intention-to-treat approach. Missing values of outcome variables and covariates will be replaced using multiple imputation procedures with 10 sets of imputations assuming missing at random. All analyses will be 2-tailed with a significance level of 2.5%.

6.3. Data management

6.3.1. Source Data

The source documents are all information collected from subjects. This includes paper CRFs, electronic CRFs and electronic spreadsheets. All paper documents will be stored safely in confidential conditions, such as lockable safe/filling cabinets.
Most subject data is collected using REDCap. REDCap is a web application for building and managing online databases.[15] Data stored within REDCap, information pertaining to the identity and activities of REDCap users are well protected. REDCap is installed under the IT infrastructure and environment in PolyU. Both the web server and database server are located behind the firewall of PolyU. Multi-factor authentication is required for the users to gain access to REDCap. Users will need to connect to a dedicated virtual private network using Microsoft multi-factor authentication pre-registered and installed in their smartphone, and then enter use a different set of login and password to login REDCap. Each user has their own account for the REDCap access to the project. Limited access to various functionality and modules such as being able to export data, to enter data or to modify the user privileges could be set up according to the role of investigators. Data Access Group could be implemented to segregate users in PolyU so the users will only gain access to necessary items to protect the privacy of the subject.
Access to the REDCap will be limited to study staff only. Anonymized patient identifiers will be used on all trial-specific documents, other than the signed consent forms and electronic folder of the participant (only available to the unmasked Investigators). The subjects will be referred to by unique registration numbers, not by name.

6.3.2. Data recording and record keeping

Direct access will be granted to authorized study staff from PolyU. All examination data will be collected by research optometrists and administrative personnel and recorded directly onto the CRFs in REDCap. If examination printouts are required to be uploaded to REDCap, researchers need to remove the subject's name and identifying information, and only write down the subject's unique study ID.

Each CRF should be completed within 3 working days of the subject visit. Data verification is conducted by unmasked researchers within 7 working days of CRFs completion.

Data will be backed-up onto a secure central cloud server. Only authorized, trained personnel will have access to the electronic data. The subjects will be identified by a unique registration number and/or code in the spreadsheet. The name and any other identifying detail will not be included in any electronic files.

Standard procedures for data management are prescribed in the Manual of Procedures.

7. Ethical approval and Consent

7.1. Human ethics approval

Study protocol, participant information sheet and consent form have received approval from the Human Subjects Ethics Sub-committee (HSESC) of the Institutional Review Board (IRB) of PolyU (HSEARS20221223001).

7.2. Informed consent/assent

Prior to or at the very beginning of the baseline visit, subjects and parents/guardians shall be fully informed of the details of research study participation.

Informed consent must be obtained from parents/guardians. Researchers shall ensure that the parents/guardians fully understand the purpose of the research study, the potential risks and benefits, the study procedures and duration, and any alternative options available.

Informed assent is obtained from subjects (child) as it is used specifically for persons who are not able to give legal consent to participate in the study. The age range for informed assent ranges from 7 to 18 years old typically. If the child is under 7 years old and lacks cognitive ability to provide informed assent, the parents/guardians will

be asked to provide informed consent on their behalf. While the child may not be able to provide informed assent, researchers shall make effort to explain the research study in age-appropriate language and help the child understand the information. It is important to involve the child in the decision-making process and to ensure they feel comfortable and safe throughout the study.

The consent/assent forms must be signed and dated by the parents/guardians and/or subjects as required and by the researchers obtaining the consents/assents. One copy of the signed consent/assent form will be given to parents/guardians.

A contact number will be provided to parents/guardians if they wish to inquire or obtain more information about the research study. The responsible research staff are Dr. Shanica Hon or Ms. Daisy Leung at Tel/WhatsApp: xxxx xxxx during business hours (9:30 A.M. to 5:45 P.M., Monday through Saturday, except on public holidays).

Standard procedures for obtaining consents/assents are described in the Manual of Procedures.

8. Assessment of Safety/Adverse Event Reporting

8.1. Definitions

8.1.1. Adverse event (AE)

An Adverse event (AE) is any untoward medical occurrence, unintended disease or injury, or untoward clinical signs (including abnormal laboratory findings) in a clinical investigation subject administered a pharmaceutical product and which does not necessarily have to have a causal relationship with the treatment.

AEs can be:

1. A new disease or symptom that was not present at baseline (enrolment)
2. An increase in the severity or frequency of a pre-existing symptom or condition
3. Abnormal laboratory test results

8.1.2. Serious Adverse Event (SAE)

A serious adverse event (SAE) is an adverse event that leads to any of the following:

1. Results in death

2. Is life-threatening, or places the participant at immediate risk of death from the event as it occurred

3. Requires hospitalization or prolongs an existing hospitalization,

4. Causes persistent or significant disability or incapacity

5. Results in congenital anomalies or birth defects

6. Is another condition that investigators judge to represent significant hazards

7. Planned hospitalization for a pre-existing condition, or a procedure required by the investigational plan, without serious deterioration in health, is not considered a serious adverse event.

8.1.3. Adverse device effect (ADE)

An Adverse Device Effect (ADE) is an adverse event related to the use of an investigational medical device.

1. This definition includes adverse events resulting from insufficient or inadequate instructions for use, deployment, implantation, installation, or operation, or any malfunction of the investigational medical device.

2. This definition includes any event that is a result of an user error or intentional abnormal use of the investigational medical device.

3. The definition includes the 'comparator' if the comparator is a medical device.

8.1.4. Serious adverse device effect (SADE)

A Serious Adverse Device Effect (SADE) is an Adverse Device Effect that has resulted in any of the consequences characteristic of a Serious Adverse Event.

8.1.5. Unanticipated problem (UP)

It is an adverse event or adverse device effect was not previously identified in nature, severity, or degree of incidence in the investigational plan, Investigator's brochure or protocol.

8.1.6. Device deficiency

Device deficiency is any inadequacy of a medical device concerning its identity, quality, durability, reliability, safety or performance:

1. Includes malfunctions, use errors, and inadequacy in the information supplied by the manufacturer including labelling.[16]
2. Includes device deficiencies related to the investigational medical device or the comparator.[16]

Adverse Events (AEs), Serious Adverse Events (SAEs), Adverse device effects (ADEs), Serious adverse device effects (SADEs), unanticipated problem (UP) and device deficiency for the research study will be recorded through REDCap.

8.2. Classification of an adverse event and adverse device effect

8.2.1. Causality

Worsening of visual acuity, development of visual disturbances (such as diplopia, ghosting, glare), eye strain symptoms, symptoms of eye infection or allergic responses (such as redness, pain, decreased vision, discharge, increased light sensitivity) are to be noted onto REDCap. An adverse event may or may not be causally related to the study intervention. The relationship of each adverse event to the trial treatment must be determined by a clinically qualified individual according to the following definitions:

- **Related:** The adverse event follows a reasonable temporal sequence from trial treatment. It cannot reasonably be attributed to any other cause.
- **Not Related:** The adverse event is probably produced by the participant's clinical state, by other modes of therapy administered to the participant or is otherwise unrelated to the trial treatment.

8.2.2. Severity

All AEs and other incidents occurring during the trial that are observed by study personnel or reported by the participant will be recorded onto REDCap, whether attributed to trial treatment. The following information will be recorded: description, date of onset and end date, severity, assessment of relatedness to trial intervention, other suspect drug or device and action taken. Follow-up information should be provided as necessary. The severity of events will be assessed on the following scale:

- **Mild:** Events require minimal or no treatment and do not interfere with the participant's daily activities.
- **Moderate:** Events result in a low level of inconvenience or concern with the therapeutic measures. Moderate events may cause some interference with functioning.
- **Severe:** Events interrupt a participant's usual daily activity and may require systemic drug therapy or other treatment. Severe events are usually potentially life-threatening or incapacitating. Of note, the term "severe" does not necessarily equate to "serious"

8.3. Responsibility

In accordance the guideline for good clinical practice (GCP) by the International Council for Harmonization of Technical Requirements for Pharmaceuticals for Human Use (ICH),[17] the Investigator/institution should ensure that adequate medical care is provided to a subject for any adverse events, including clinically significant laboratory values, related to the trial. The Investigator/institution should inform a subject when medical care is needed for an intercurrent illness of which the Investigator becomes aware. The unmarked Investigator and the PI need to evaluate the AE severity as well as the relatedness to the study procedures and study device.

8.4. Reporting

Related Serious adverse events must be reported to the IRB and the sponsor of the study by the Investigator as soon as possible, the IRB of the Hong Kong Polytechnic University requires related SAE be reported within 48 hours. ICH[17] and the U.S. Food and Drug Administration[18] require fatal SAE be reported within 7

calendar days while non-fatal SAE be reported within 15 calendar days. If a serious related and unexpected AE represents an increased risk to the subject, or affects willingness to continue in the study, that information will be provided to the subject(s) and parent/guardian(s) through an update to the consent/assent, approved by the IRB, as soon as possible.

8.5. Documentation and follow-up

At each study visit, the Investigator or designee will inquire about the occurrence of AEs/SAEs since the last visit. Any AE observed by the Investigator or reported by the subjects will be documented on an adverse event form onto REDCap as soon as possible after notification of the event. However, not all AEs require reporting, as they might not directly impact participant risk. Information to be collected includes event description, time of onset, clinician's assessment of severity, relationship to study product (assessed only by those with the training and authority to make a diagnosis), and time of resolution/stabilization of the event. All AEs occurring while on study must be documented appropriately regardless of relationship. All AEs will be followed to adequate resolution. An appointment will be arranged for 1-4 weeks following AE resolution to check for any recurrence or changes because of the AE. Changes in the severity of an AE will be documented to allow an assessment of the duration of the event at each level of severity to be performed. AEs characterized as intermittent require documentation of onset and duration of each episode. These will be followed-up and assessed by medically fit clinician. It will be left to the judgement of the research team to decide whether an AE is of sufficient severity to require discontinuing the participant from the study. A subject may also voluntarily withdraw from participating in the study due to what he or she perceives as an intolerable AE. If either of these occurs, the subject must undergo follow-up visits for trial assessment and be given appropriate care under medical supervision until symptoms cease, or the condition becomes stable.

8.6. Unmaking

This is a double-masked trial, where both subjects and Investigators are masked to treatment allocation. Unmasking will only occur at the end of the study under the authorization of the project co-ordinator.

For individual emergency unmasking, where a related serious adverse event has occurred and the group allocation of the subject is required in order to enable clinical treatments to be planned, project co-ordinator will inform the allocation of the subject, and this will be documented. The masked Investigator will remain masked.

8.7. Data safety and monitoring

Ellenberg et al.[19] provide guidelines for deciding whether a data safety monitoring committee (DSMC) needs to be established for a trial. They propose that if two or more of the following criteria are met then a DSMC is required. 1) The trial is intended to provide definitive information about the effectiveness and/or safety of a medical intervention. 2) There is prior data to suggest that the intervention being studied has the potential to induce potentially unacceptable toxicity. 3) The trial is evaluating mortality or another major endpoint such that inferiority of one treatment arm has safety as well as effectiveness implications. 4) It would be ethically important for the trial to stop early if the primary question addressed has been definitively answered, even if secondary questions or complete safety information were not fully addressed.

This trial meets only point 1 indicating that an independent DSMC does not need to be established for the trial. However, data safety and regular monitoring will be performed by the research team. Compliance with the protocol and accuracy in relation to source documents will be evaluated.

An independent monitor will check the existence and correct date for all signed consent forms. The monitor will check data collection and data entry for all recruited subjects. The monitor will confirm collected data on hard copy printouts (the source data) and check the data entry from the hard copy to REDCap.

9. Intervention supplies

9.1. Study treatment identification

The following products will be used for intervention in this study:

Products	Supplier

Single Vision spectacle lenses	Hoya Lens Thailand Limited
D1 and D2 spectacle lenses	Hoya Lens Thailand Limited

9.2. Handling and dispensing of study treatment

All spectacles used in this study are maintained under the direct responsibility of the project coordinator. It will be the project coordinator's responsibility to ensure that an accurate record of the interventions issued to subjects is maintained. All spectacles and lenses, whether worn or not worn, will be returned to the project coordinator on withdrawal/discontinuation from the study.

9.3. Lens verification, packaging and labelling

When the spectacle lenses arrive from the supplier, they will be verified by the project coordinator. The spectacle lenses are packaged with lens case and lens cloth with unique subject ID labelled on the lens case.

9.4. Treatment supply records

It will be the responsibility of the project coordinator to ensure that an inventory of the interventions is maintained. REDCap Records will include:

- Amount and record of spectacles used in the study
- Unique participant identifier (when allocated)
- Initials of the person who dispensed the interventions

10.Study completion

All investigational spectacles (used or unused) will be returned to the research staff after completion of the final data collection visit. As a token of appreciation, each participant will receive either a pair of SV spectacles or MiyoSmart spectacles upon completion of the study.

As a double-masked RCT, subjects and parents/guardians are masked from the group allocation and study intervention until the data analysis is completed. After completion of study, they will receive a thank-you letter through email or WhatsApp

for participating in the study. The group allocation of subjects will also be disclosed in the thank-you letter.

11.Dissemination of results

11.1. Study subjects

All the subjects will receive a letter of thanks for participating in the study, a summary of the study results, and an outline of their significance when the study is complete.

11.2. Academic/professional colleagues

Articles detailing the trial results will be submitted to leading international peer-reviewed journals and data will be presented at international conferences.

12.Administrative Section

12.1. Adherence to the protocol

The approved protocol will be strictly followed throughout the study. Exceptions will only apply to eliminating an immediate hazard to participants. Any protocol deviation will be documented in the protocol violations form (recorded in electronic spreadsheet).

12.2. Protocol revision procedures

Any amendment will be submitted by the PI to the IRB of PolyU for review and approval or favourable opinion prior to implementation. Documentation of approval signed by the chairperson or designee of the IRB and sent to the PI will be filed.

Procedures listed below will be followed if an amendment substantially alters the study design or increases the potential risk to the subject:

- The consent form will be revised and submitted to the IRB for review and approval or favourable opinion;
- If the current enrolled participants are affected by the amendment, the subjects will first be contacted by telephone, the amendment will be discussed, and verbal consent will be re-obtained;
- Furthermore, the revised consent form will be sent to the current enrolled subjects by post.

12.3. Case report form procedures

Data collection will be directly inputted into CRFs in REDCap. Information recorded onto paper CRFs will be entered onto REDCap as soon as possible. The paper CRFs will be filed in the subjects' study folder. All paper CRFs will be completed legibly in pen by the research staff at the time of each assessment. Corrections will be made in the following manner: incorrect entry will be corrected using a red pen with a single strikethrough to ensure that the previous entry is still legible; the correction will be initialed and dated by the person who makes the correction. Once the paper form is completed, it will be entered onto REDCap by the same research staff as soon as possible.

CRFs containing subjects' identifiable details will be locked with other identifiable material and this information will not be entered onto REDCap or released from the study centre.

12.4. Monitoring/Source document verification

Data collection and data entry onto CRFs and the study spreadsheet will be monitored closely and regularly. The monitor will review all registered subjects' records to ensure that they have provided informed consent. The monitor will review the study documentation and records to ensure that

1) all documentation is up to date, including the correct version of the Study Protocol and Manual of Procedures;
2) and the record-keeping meets the requirements specified in the protocol.

The monitor will also audit that the product supply records are maintained and that there are sufficient supplies remaining. The handling process will also be monitored against the study procedure.

12.5. Data confidentiality and security

All CRF datasheets and consent forms collected from the subjects will be treated as confidential and stored securely at each study site. The secure cloud server and subject records will be accessed only by the researchers involved in the study. REDCap employs various methods to protect against malicious users who attempt to identify and exploit the security vulnerabilities in REDCap. Processes such as sanitization, filtering, data type checking and escaping are implemented to help protect against methods of attack.

Published data will not contain subject names. All the participants will only be identified by code when data is sent outside of the institution.

The study PI, Dr Dennis Yan-yin Tse, will be responsible for all personal information regarding the research study.

12.6. Reporting schedule

The project coordinator will provide annual reports of study progress to the IRB of PolyU and the sponsor of the study. All related SAEs that are fatal or life-threatening and suspected of being related to the treatment used in the trial will also be reported within 48 hours.

12.7. Record retention policy

The PI shall ensure that PolyU retains all information and documentation related to the Project for five years from the date of the final report. PolyU shall exercise necessary supervision to ensure that such records and documentation are appropriately and confidentially maintained for three (3) years from the date of provision in the case of providing information, and for five (5) years from the date the end of the said research is reported in the case of receiving specimens and/or information. Staff involved in the study will not destroy any record associated with the trial without prior approval from the PI.

If any co-investigators withdraw from the study (e.g. relocation or retirement), any records they hold will be transferred to a mutually agreed upon designee, such as another co-investigator. Notice of such transfer will be given in writing.

12.8. Record Disposal

For the disposal of study materials and confidential personal documents, 5 years from the date of the final research report, we will utilize the services of a local company called Secure Information Disposal Services Limited,[20] who specialize in confidential document destruction.

12.9. Insurance

Subjects in this clinical trial are covered under PolyU's Master Clinical Trial Insurance.

12.10. Dissemination/Publicity Method

The study's deliverable is co-owned by PolyU and Hoya. With agreement from Hoya, the Investigators may publish or present the results of this study at conferences. Potential secondary use of research data within the Optometry Research Clinic of PolyU, such as sharing with other research studies to enhance scientific knowledge and contribute to further advancements in the field, may be conducted upon agreement from Hoya. No data transfer will be allowed to party other than those mentioned above.

PolyU shall submit any planned publications, including theses, dissertations, specialist publications and conference contributions, to the sponsor at least four weeks before the intended date of publication. Insofar as publication is impaired by the sponsors' legitimate interests, publication will only take place once the parties have reached an understanding on the wording.

13. Abbreviations

AE	Adverse event
ADE	Adverse device effect
AL	Axial length
BCVA	Best-corrected visual acuity
Co-I	Co-Investigator
CRF	Case Report Form
D1	Defocus Incorporated Multiple Segments spectacle lenses
D2	Second generation of DIMS
DIMS	Defocus Incorporated Multiple Segments
DSMC	Data safety monitoring committee
EKC	Epidemic Keratoconjunctivitis
ETDRS	Early Treatment Diabetic Retinopathy Study
GCP	Good clinical practice
HSESC	Human Subjects Ethics Sub-committee
ICH	International Council of Harmonization
ID	Identifications
IOP	Intraocular pressure
IRB	Institutional Review Board
logMAR	Logarithm of the Minimum Angle of Resolution
MOP	Manual of procedures
OCT	optical coherence tomography
PolyU	Hong Kong Polytechnic University
PI	Principal Investigator
REDCap	Research Electronic Data Capture
RCT	Randomised clinical trial
RPR	Relative peripheral refraction
Rx	Prescription
SADE	Serious adverse device effect
SAE	Serious adverse event
SER	Spherical equivalent refraction
SICS	Solution induced corneal staining
ssOCT	swept source optical coherence tomography
SV	Single vision
UP	Unanticipated problem
VA	Visual acuity

14.Reference

1. Yam JC, Tang SM, Kam KW, et al. High prevalence of myopia in children and their parents in Hong Kong Chinese Population: the Hong Kong Children Eye Study. Acta Ophthalmol. 2020;98:e639-648.
2. Fan DS, Lam DS, Lam RF, et al. Prevalence, incidence, and progression of myopia of school children in Hong Kong. Invest Ophthalmol Vis Sci. 2004;45:1071–1075.
3. Holden BA, Fricke TR, Wilson DA, et al. Global Prevalence of Myopia and High Myopia and Temporal Trends from 2000 through 2050. Ophthalmology. 2016;123:1036-42.
4. Grødum K, Heijl A, Bengtsson B. Refractive error and glaucoma. Acta ophthalmologica Scandinavica. 2001;79:560-6.
5. Wong TY, Klein BE, Klein R, et al. Refractive errors, intraocular pressure, and glaucoma in a white population. Ophthalmology. 2003;110:211-7.
6. Vongphanit J, Mitchell P, Wang JJ. Prevalence and progression of myopic retinopathy in an older population. Ophthalmology 2002;109:704–11.
7. Saw SM, Gazzard G, Shih-Yen EC, et al. Myopia and associated pathological complications. Ophthalmic & physiological optics : the journal of the British College of Ophthalmic Opticians (Optometrists). 2005;25:381-91.
8. Holden B, Sankaridurg P, Smith E, et al. Myopia, an underrated global challenge to vision: Where the current data takes us on myopia control. Eye 2014;28:142–6.
9. Fricke TR, Holden BA, Wilson DA, et al. Global cost of correcting vision impairment from uncorrected refractive error. Bull World Health Organ 2012;90:728–38.
10. Huang J, Wen D, Wang Q, et al. Efficacy Comparison of 16 Interventions for Myopia Control in Children: A Network Meta-analysis. Ophthalmology. 2016;123:697-708.
11. Lam CSY, Tang WC, Tse DY, et al. Defocus Incorporated Multiple Segments (DIMS) spectacle lenses slow myopia progression: a 2-year randomised clinical trial. The Br J of Ophthalmol. 2020;104:363-8.
12. Chen Y, Zhang J, Morgan IG, et al. Identifying children at risk of high myopia using population centile curves of refraction. PloS One 2016;11:e0167642.
13. Pan Y, Tarczy-Hornoch K, Cotter SA, et al. Visual acuity norms in pre-school children: the Multi-Ethnic Pediatric Eye Disease Study. Optometry and vision

science: official publication of the American Academy of Optometry. 2009;86:607-12.
14. Anstice NS, Thompson B. The measurement of visual acuity in children: an evidence-based update. Clinical & experimental optometry. 2014;97:3-11.
15. Harris PA, Taylor R, Thielke R, et al. Research electronic data capture (REDCap)--a metadata-driven methodology and workflow process for providing translational research informatics support. J Biomed Inform. 2009;42:377-81.
16. International Organization for Standardization (ISO). ISO 14155:2011 - International GCP standard for medical device clinical trials. [Internet]. [cited 2022 Oct 29]. Available from: https://www.iso.org/obp/ui/#iso:std:iso:14155:ed-3:v1:en
17. The International Council for Harmonisation of Technical Requirements for Pharmaceuticals for Human Use (ICH). ICH Harmonised Tripartite Guideline. The Addendum to ICH E6(R1): Guideline for good clinical practice E6(R2). [Internet]. [cited 2022 Oct 29]. Available from: https://database.ich.org/sites/default/files/E6_R2_Addendum.pdf
18. U.S. Food and Drug Administration. IND Application Reporting: Safety Reports. [Internet] [cited 2022 Dec 19]. Available from: https://www.fda.gov/drugs/investigational-new-drug-ind-application/ind-application-reporting-safety-reports
19. Ellenberg SS, Culbertson R, Gillen DL, et al. Data monitoring committees for pragmatic clinical trials. Clin Trials. 2015;12:530-536.
20. Secure Information Disposal Services Limited. Accessed Jul 24, 2023. https://en.ssid.hk/
